# Analysis of area level and unit level models for small area estimation in forest inventories assisted with LiDAR auxiliary information

**DOI:** 10.1371/journal.pone.0189401

**Published:** 2017-12-07

**Authors:** Francisco Mauro, Vicente J. Monleon, Hailemariam Temesgen, Kevin R. Ford

**Affiliations:** 1 Oregon State University, College of Forestry, Forest Engineering Resources and Management Department, Corvallis, Oregon, United States of America; 2 US Forest Service, Pacific Northwest Research Station, Forestry Sciences Laboratory, Corvallis, Oregon, United States of America; 3 Bureau of Land Management Oregon/Washington State Office, Portland, Oregon, United States of America; University of the Chinese Academy of Sciences, CHINA

## Abstract

Forest inventories require estimates and measures of uncertainty for subpopulations such as management units. These units often times hold a small sample size, so they should be regarded as small areas. When auxiliary information is available, different small area estimation methods have been proposed to obtain reliable estimates for small areas. Unit level empirical best linear unbiased predictors (EBLUP) based on plot or grid unit level models have been studied more thoroughly than area level EBLUPs, where the modelling occurs at the management unit scale. Area level EBLUPs do not require a precise plot positioning and allow the use of variable radius plots, thus reducing fieldwork costs. However, their performance has not been examined thoroughly. We compared unit level and area level EBLUPs, using LiDAR auxiliary information collected for inventorying 98,104 ha coastal coniferous forest. Unit level models were consistently more accurate than area level EBLUPs, and area level EBLUPs were consistently more accurate than field estimates except for large management units that held a large sample. For stand density, volume, basal area, quadratic mean diameter, mean height and Lorey’s height, root mean squared errors (*rmses*) of estimates obtained using area level EBLUPs were, on average, 1.43, 2.83, 2.09, 1.40, 1.32 and 1.64 times larger than those based on unit level estimates, respectively. Similarly, direct field estimates had *rmse*s that were, on average, 1.37, 1.45, 1.17, 1.17, 1.26, and 1.38 times larger than *rmses* of area level EBLUPs. Therefore, area level models can lead to substantial gains in accuracy compared to direct estimates, and unit level models lead to very important gains in accuracy compared to area level models, potentially justifying the additional costs of obtaining accurate field plot coordinates.

## 1 Introduction

To make management decisions, forest planning requires information at different scales. This information is typically obtained through sampling, which implies a certain level of uncertainty. For some scales or subpopulations, the sample size may be too small, and the uncertainty too large, to meet the information needs. While the uncertainty can be reduced by increasing the sample size in the subpopulations of interest, this solution may not be efficient or affordable, as increasing the sample size would increase the cost of fieldwork beyond acceptable limits. Estimation techniques that use auxiliary information usually allow for gains in efficiency when compared to forest inventories based on plot information alone [1,2]. However, even then, efficiency gains may not be sufficient to meet the information needs for very small subpopulations. Small area estimation (SAE) is a set of model-based techniques for predicting the variables of interest for subpopulations with very few or even no observations. Typically, they combine predictions from a model based on observations from the entire population (synthetic prediction) with estimators derived from the observations from the subpopulation of interest (direct estimation) [3].

Forest inventories often aim to provide estimates and measures of uncertainty for variables in management units (MU) such as stands, harvest and treatment units, districts, or even larger areas like municipalities or counties. Those MUs tend to be relatively homogeneous internally, but differ in age, site quality or forest structure. When the MUs are large enough to hold a relatively large sample, they can be treated as strata and either design-based estimators or stratum-specific models can be developed for each of them separately. In this case, the between MU variability is accounted for by separately deriving MU specific models, estimators and measures of uncertainty that do not consider any information from MUs other than the one under consideration. However, in practice, a more common situation is that MUs only have a small sample size, so that MU specific modeling and estimation is not possible. Then, MUs can be regarded as small areas and estimation can be based on models that include information from multiple MUs and explicitly account for the between-MU variability [4]. However, model based estimation techniques rely on specifying a correct model to generate unbiased estimators of the variable of interest and their uncertainty. Empirical best linear unbiased predictors (EBLUPs) based on linear mixed effect models that include auxiliary information and MU random effects are one of the most common approaches in the small area estimation literature [5] and have been successfully applied to forest inventories [6–9]. Two different types of EBLUPs can be found in the SAE literature, depending on the modeling scale: unit level and area level models.

For unit level models, the study area is covered by a grid where each cell is considered a population unit with known values of the auxiliary variables [10]. Both the response and the auxiliary variables are measured in a sample of field plots of size similar to that of the grid cells. This sample is used to fit models that relate the variable of interest to the auxiliary information and that include MU random effects. The variable of interest is then predicted at levels that can range from single grid units to any combination of grid units, including full or partial MUs and up to the entire study area [9]. Unit level EBLUPs were introduced by Battese, Harter and Fuller [11] and were first used in forest inventories by Breidenbach and Astrup [7] and Goerndt, Monleon and Temesgen [8].

Area level models operate at the MU level, relating MU specific auxiliary information to direct estimates of the variables of interest. The auxiliary information and the models are derived at a much coarser scale, because MUs are typically orders of magnitude larger than plots or grid units. For the typical forest variables of interest (e.g. volume, basal area or stand density), the predictive power of the MU specific auxiliary information and associated models can be expected to be lower than the predictive power of the auxiliary information used in unit level models, which is collected at the plot level. However, despite their potentially lower predictive performance, area level models are very appealing for practical applications because, for unit level models, the auxiliary information must be known accurately for each field plot while, for area level models, it is only necessary to know to what MU each field observation belongs. This property of area level models eliminates the need for precise field plot coordinates, simplifies the fieldwork load, and allows the use of auxiliary information in combination with inventories based on variable radius plots. An additional consideration is that, for unit level models, the areas of interest can consist of single pixels or groups of pixels, possibly spanning parts of several MUs, allowing for a high degree of flexibility in how these models can be used for prediction. However, for area level models, inference is only possible for entire MUs or groups of MUs. Area level models were first used by Fay and Herriot [12] and introduced in the forest inventory and forest fire modelling literature by Goerndt, Monleon and Temesgen [6] and Boubeta et al. [13].

While the possibility of using small area estimation methods that do not require knowing the exact plot coordinates makes area level models a very appealing alternative to unit level models, no study to date has directly compared the two modeling techniques. A direct comparison of EBLUPs based on area level versus unit level models is still necessary, as it would provide valuable insights to assess whether the lower fieldwork requirements of area level models can outweigh their expectedly worse predictive performance. Therefore, the main objective of the study is to compare the performance of unit level and area level models for estimating six forest attributes (stand density (N), Volume (V), basal area (BA), quadratic mean diameter (QMD), mean height (H_m_) and Lorey’s height (H_L_)) for MUs with small sample sizes.

## 2 Material and methods

The field measurements performed in the Coos-Bay and Curry counties (44.04°N, 123.54°W, 42.09°N, 124.51°W) was approved by the US Bureau of Land Management (BLM) and did not affect any protected or endangered species (See section 2.2).

### 2.1 Study Area and Management Units (MUs)

A LiDAR survey acquired by the Oregon Department of Geology and Mineral Industries (DOGAMI) during 2008 and 2009 covered an area of approximately 1.5 million hectares in the counties of Coos and Curry, Southwestern Oregon (SWO). The study area includes the 98,104 ha of forested lands administered by the Bureau of Land Management (BLM) within the SWO DOGAMI flight. Limits to the North, East, South and West of the study area are 44.04°N, 123.54°W, 42.09°N, 124.51°W respectively. The most common vegetation type is coastal coniferous forests with a variable degree of species mixing. The dominant species is Douglas-fir (*Pseudotsuga menziesii* (Mirb) Franco), but other softwoods such as western hemlock (*Tsuga heterophylla* (Raf.) Sarg.), Sitka spruce (*Picea sitchensis* (Bong.) Carr.), and western redcedar (*Thuja plicata* Donn ex D.Don) are relatively common. The most common hardwood species are red alder (*Alnus rubra* Bong.) and bigleaf maple (*Acer macrophyllum* Pursh), but they are less common than the dominant softwood species.

The BLM continuously maintains the Forest Operations Inventory (FOI) spatial database. This database contains descriptions for delineated stands covering all the BLM-administered lands in SWO. Information about the stands in the FOI database includes stand age, an overall description of the vegetation, and a description of the different vegetation layers present in each stand. The average delineated stand size in the study area is 10.46 ha. With the available sample of 867 field plots (see section 2.2), almost 90% of the stands did not contain any field plots, and most stands in the remaining 10% contained only a single plot. Therefore, it was impossible to model the variability among individually delineated stands (i.e. obtain models with stand level random effects).

Thus, the MUs considered in this study were defined by aggregating forested stands that were in the same age class and had the same species group (determined by the dominant species present), since stand and species group have important implications for management. The age classes were those defined in the BLM’s FOI spatial database: stands of 5 years or younger, and 10-year age classes for all stands greater than 5 years (6 to 15 year old, 16 to 25 year old, 26 to 35 year old and so on). The oldest stands were 576 to 585 year old. The observed species groups were Douglas-fir, mixed-conifer and hardwood. Not all the possible combinations of species group and age classes are present in the study area. The aggregation method that we used was similar to what was used in the vegetation modeling for BLM’s Resource Management Plans for Western Oregon [14].

**Fig 1:**
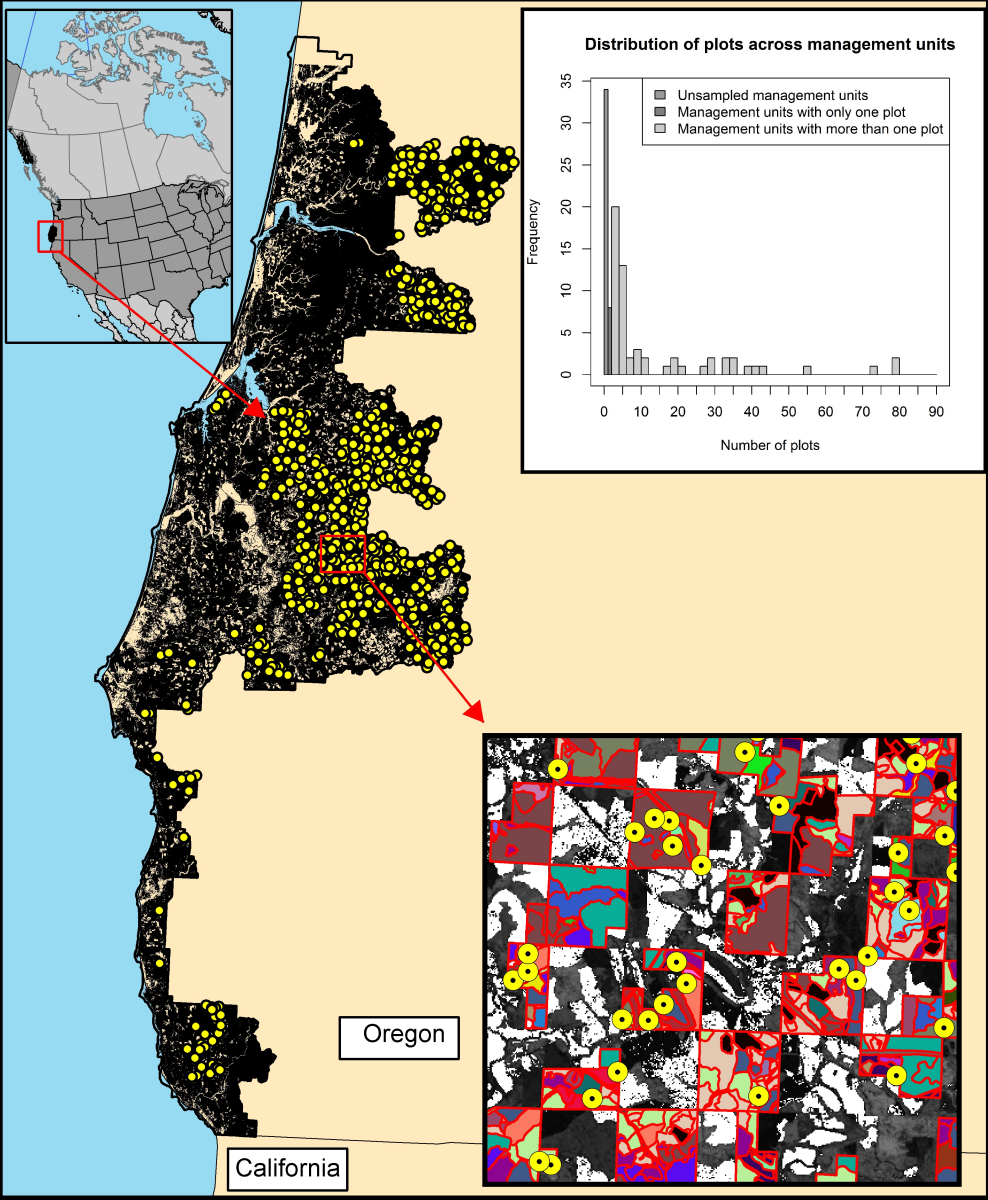
Study area and location of sample plots. General location of the study area within the US state of Oregon, location of all plots and detailed view of an example of plots and stands. A histogram of the distribution of the sample size (*n*_*i*_) across management units is shown on the upper right corner. Plots are represented by solid circles. Each management unit is represented using the same color. Stand boundaries are represented using a solid line.

We only considered MUs (age class by species group combinations) of at least 4.04 ha (10 acres). In total, there were 84 MUs larger than 4.04 ha, 64 contained at least one field plot and 58 contained at least 2 plots (Fig 1).

### 2.2 Field data collection

The study area was sampled following a probability-based sample design similar to that described in [15,16]. The BLM approved the sampling in the study area. The sample consisted of a total of 867, 0.052 ha circular plots, approximately the same size as the grid units. In each plot, the diameter at breast height (DBH), height, species, crown base height, and crown ratio of every live tree larger than 13.97 cm (5.5 in) diameter at breast height were recorded. Live trees with a diameter at breast height less than 13.97 cm were measured in smaller, concentric 0.008 ha plots. Standing volume for each tree was computed using regional species-specific volume equations included in the US Forest Service’s National Volume Estimator Library [17]. All variables were aggregated to the plot level by applying the corresponding expansion factors to large (DBH≥ 13.97 cm) and small (DBH< 13.97 cm) trees and transformed to per hectare values when appropriate.

Field crews located the plot center using coarse acquisition code global navigation satellite system. Phase observations were recorded by leaving the receiver on the plot center for at least 10 minutes. Those observations were post processed, so that location errors after differential corrections are expected to be negligible.

### 2.3 LIDAR collection, processing and computation of model predictors

LIDAR data were collected during the spring and summer of 2008 and 2009 using a Leica LIDAR Phase II laser. The average return density was 8.1 returns/m^2^, the average flying altitude was 900m above sea level, and the field of view was 28° (±14° from nadir). Additional details about the LiDAR flight survey can be found in [16].

A square grid with a cell size of 22.86 m (0.052ha) was overlaid on the study area and each cell represented a unit in the unit level analysis. A set of 44 predictors derived from the LIDAR data and the FOI database were obtained for each grid cell and for each field plot. In total, 28 predictors summarizing the most relevant information about the point cloud of each grid cell were derived using FUSION [18], including extreme values, percentiles, and fractions of pulses above different thresholds. Two additional predictors, the MU age and dominant species (a categorical variable) were added from the BLM FOI database. Finally, the LIDAR data was used to obtain a digital terrain model (DTM), a digital surface model (DSM), and a digital canopy height model (DCHM). Slope and aspect were derived from the DTM. All these five layers had a grid cell size of 1 meter. Their values were aggregated for each plot and unit of the LIDAR grid for a total of 14 additional variables. A detailed description of the unit level predictors and their computation is included in S1 Appendix.

For the area level models, a set of 86 predictors was obtained. Area level predictors were grouped into four sets depending on how they were obtained. The first group consisted of approximate quantiles of the height distribution of returns within each MU. The second group contained MU averages and variances of unit level predictors summarizing the point cloud of each unit. The third group was the age and dominant species of each MU recorded in the FOI database, and the fourth group consisted on the same predictors that were derived from units using the 1 m resolution DTM, DCHM, slope and aspect layers. A detailed description of the area level predictors and their computation is included in S1 Appendix.

### 2.4 Unit level models

The general form of the unit level models was (Eq(1)):

$$y_{ij}=x_{ij}^{t}\beta +v_{i}+e_{ij}$$ (1)

where the subscript *i* = 1,…,*m* (*m* = 84) indexes the MU and *j* = 1,…,*N*_*i*_ indexes the population unit (grid cells or plots) within the MU. The terms *y*_*ij*_, $x_{ij}^{t}$ and *e*_*ij*_ are the value of the response variable, the transpose of the vector of predictors, and the model errors for the *j*^*th*^ population unit within the *i*^*th*^ MU. The MU random effects are assumed to be independently distributed $v_{i}∼N(0,\sigma_{v}^{2})$ and independent of the model errors *e*_*ij*_. Heteroscedasticity of model errors was allowed, assuming that $e_{ij}={\sigma_{e}mcp}_{ij}^{\eta }\epsilon_{ij}$, where *mcp*_*ij*_ is the predictor most correlated to the variable of interest. Under this formulation, *ϵ*_*ij*_ is the standardized error, distributed as *ϵ*_*ij*_ ∼ *N*(0,1), so that ${V(e_{ij})}^{1/2}=\sigma_{e0}{mcp}_{ij}^{\eta }$, and *η* is a model parameter with possible values equal to 0, 0.5 and 1. When heteroscedasticity was not present, the parameter *η* was 0 and the term ${mcp}_{ij}^{\eta }$ was 1.

### 2.5 Area level models

The basic area level model, also known as the Fay-Herriot model, [12] arises from the combination of a sampling model and a regression model linking the parameter of interest for each MU to the predictors. The parameter of interest for the *i*^*th*^ MU is the mean of the variable of interest, denoted as *μ*_*i*_. It is linked to the auxiliary information through the regression model (2)

$$\mu_{i}=w_{i}^{t}\beta +v_{i}$$ (2)

where *v*_*i*_ is assumed to be normally distributed with mean 0 and variance $\sigma_{v}^{2}$ and ***w***_*i*_ is a vector of predictors computed at the MU level.

A direct estimator of *μ*_*i*_, based only on the sample of plots within the MU, is available and it is denoted as ${\hat{\mu }}_{Field\;i}$. In this case ${\hat{\mu }}_{Field\;i}$ is the mean of the plots within a MU. The subscript Field is used to emphasize that the direct estimator is computed using only field information and no auxiliary information is considered for its computation. Both *μ*_*i*_ and ${\hat{\mu }}_{Field\;i}$ are related through the sampling model (3)

$${\hat{\mu }}_{Field\;i}=\mu_{i}+e_{i}$$ (3)

where *e*_*i*_ is the error of the direct estimator ${\hat{\mu }}_{Field\;i}$ for the *i*^*th*^ MU, which is assumed to be normally distributed with mean 0 and variance $\sigma_{e\;i}^{2}$. The direct estimator ${\hat{\mu }}_{Field\;i}$ in this case is the sample mean of the variable of interest for the field plots within the *i*^*th*^ MU. When the sampling model and the regression model are combined, we obtain model (4).

$${\hat{\mu }}_{Field\;i}=w_{i}^{t}\beta +v_{i}+e_{i}$$ (4)

### 2.6 Estimators and their MSE

The parameters of interest were the MU means of N, V, BA, QMD, H_m_ and H_L_. For a given variable of interest, the target parameter will be denoted as *μ*_*i*_, where *i* is used to index the MU. The direct estimates of *μ*_*i*_ obtained using only the field information, ${\hat{\mu }}_{Field\;i}$, were used as a reference to evaluate potential gains in efficiency due to the inclusion of the LIDAR auxiliary information through either area level model or unit level models. The direct estimator is the mean of the plot measurements within each MU, (${\hat{\mu }}_{Field\;i}$). The estimator of its mean square error (MSE) for unit *i* is $mse({\hat{\mu }}_{Field\;i})\;=\;\frac{1}{n_{i}}\sum_{j\;=\;1}^{n_{i}}\frac{{\left(y_{ij}-{\hat{\mu }}_{Field\;i}\right)}^{2}}{n_{i}-1}$, of the root mean square error (RMSE) is $rmse({\hat{\mu }}_{Field\;i})\;=\;\sqrt{mse({\hat{\mu }}_{Field\;i})}$ and of the coefficient of variation (CV) is $CV(\mu_{Field\;i})\;=\;\frac{rmse({\hat{\mu }}_{Field\;i})}{{\hat{\mu }}_{Field\;i}}$. These estimators were only computed for MUs with two or more plots.

For each SAE method and variable of interest, a model relating the LIDAR auxiliary information and field information was selected (see S2 Appendix for details on the model selection procedure). After model selection, we estimated the parameter of interest for each MU from the unit level model (${\hat{\mu }}_{Unit\;i}$) and the area level model (${\hat{\mu }}_{Area\;i}$). We also estimated the mean square error, root mean square error and coefficient of variation as detailed in S2 Appendix.

### 2.7 Comparison of estimation methods

We compared estimates and measures of uncertainty derived from area level models, unit level models and direct estimators using only field data.

First, for each variable and for each MU, the three methods were sorted according to their CV and *rmse* and the best method chosen. Then, considering only the MUs where the sample size was larger than 2 plots, so that it was possible to compare the three methods, we calculated the proportion of study area for which each method was best.

Then, we compared the estimates and measures of uncertainty between: 1) area level estimates against unit level estimates (Area vs Unit), 2) field estimates against area level estimates (Field vs Area) and 3) field estimates against unit level estimates (Field vs Unit). For each comparison, we analyzed the agreement of the MU estimates obtained with each method by computing the differences between ${\hat{\mu }}_{Unit\;i}$ and ${\hat{\mu }}_{Area\;i},\;{\hat{\mu }}_{Field\;i}$ and ${\hat{\mu }}_{Area\;i}$ and ${\hat{\mu }}_{Field\;i}$ and ${\hat{\mu }}_{Unit\;i}$. We evaluated the increase/reduction in the estimated uncertainty when using one method instead of another using root mean square error ratios (*rmser*) in Eqs (5), (6) and (7). Average *rmser* values for each pair of methods were computed by weighting each MU ratio by the MU area.

$${rmser}_{Area\;Unit\;i}=\frac{rmse({\hat{\mu }}_{Area\;i})}{rmse({\hat{\mu }}_{Unit\;i})}$$ (5)

$${rmser}_{Field\;Area\;i}=\frac{rmse({\hat{\mu }}_{Field\;i})}{rmse({\hat{\mu }}_{Area\;i})}$$ (6)

$${rmser}_{Field\;Unit\;i}=\frac{rmse({\hat{\mu }}_{Field\;i})}{rmse({\hat{\mu }}_{Unit\;i})}$$ (7)

#### 2.7.1 Uncertainty of estimates by MU sample size

Larger MUs contain more sample plots and the number of plots within a MU is related to the uncertainty of the estimators. To evaluate how the uncertainty and the agreement between estimates changed when changing the MU size and the associated sample size, we defined five groups of MUs depending on the number of plots that they contained. The first group consisted of 20 unsampled MUs, the second group contained 6 MUs with only one plot, the third group contained 33 MUs with 2 to 5 plots, the fourth group had 11 MUs with 6 to 25 plots and the fifth group contained 14 MUs with more than 25 plots. For each group, we calculated the proportion of the area covered by the group where each method was best in terms of *CV* or *rmse*.

## 3 Results and discussion

### 3.1 Selected models

Selected unit level models included only one or two predictors for all variables except for N (Table 1). The selected predictors were always derived from LiDAR metrics only, except for basal area where selected predictors were computed from the DTM and the DCHM. Neither MU age nor dominant species were selected in any of the final models and were included only in a few candidate models. For the area level models, the performance metrics showed a more gradual improvement as the number of predictors increased, resulting in models with more predictors than the unit level models. The models selected for V, BA, QMD and H_m_ included predictors derived from LiDAR metrics as well as predictors derived from the DTM (Table 2). As with the unit level models, the final models did not include neither MU age nor dominant species.

**Table 1:** Predictors and variance parameters for the selected unit level models. When the error variance was not constant, *mcp* was the first predictor, associated to *β*_1_. Elev_mean and Elev_sd are the mean and standard deviation of the lidar heights. Elev_mean_sq is the square of the LiDAR height. Elev_P50, Elev_P70 and Elev_P80 are the 50^th^, 70^th^ and 80^th^ percentile of the LiDAR heights. all_co_ab_2m and 1st_co_ab_mean are the proportion of returns above 2m and the proportion of first returns above the mean LiDAR height respectively. See S1 Appendix for additional details on the variables and model selection procedure.

Variable	Coefficients and auxiliary variable					*η*	*σ*_*e*_	*σ*_*v*_
	*β*_1_	*β*_2_	*β*_3_	*β*_4_	*β*_5_			
N(stems/ha)	DTM_MIN	Elev_P80	all_co_ab_2m	1^st^_co_ab_mean	Elev_P50	1	0.04	8.39
V(m^3^/ha)	Elev_mean_sq	all_1^st^_co_ab_2m				0.5	0.26	2.39
BA (m^2^/ha)	DTM_MEAN	DCHM_MEAN				0.5	69.14	84.35
QMD (cm)	Elev_P70					0.5	3.88	9.61
H_m_ (m)	Elev_mean					0.5	0.30	0.78
H_lor_ (m)	Elev_mean	Elev_sd				0	0.26	0.61

**Table 2:** Predictors and variance parameter $\sigma_{v}^{2}$ for the selected area level models. For each MU, Elev_P75_m is the mean of the 75^th^ percentile of the elevation of LiDAR returns for the pixels within the MU; Slope_m and Slope_s are the mean and variance of DTM-derived slopes; all_1st_co_2m_m and Elev_ave_sq_m are the average of the pixel level percentage of first returns above 2m and the pixel level values of the square of the mean elevation of LiDAR returns; Elev_P01_v Elev_P80_v and Elev_P99_v, are the variance of the pixel level values of the 1^st^, 80^th^ and 99^th^ percentile of the LiDAR elevations; Elev_CV_v and Elev_max_v are the variance of the pixels interquartile range, coefficient of variation and maximum of LiDAR elevation heights; 1^st^_co_ab_2m_v is the variance of the pixel level values of the percentage of first returns above 2m; Slope_s and Slope r are the standard deviation and range of the pixel level slope; A_P10, A_P20, A_P30 and A_P60_are approximate percentiles of the LiDAR return height distribution within the MU. See S1 Appendix for the details on the computation of the LiDAR predictors.

Variable	Coefficient and auxiliary variable					*σ*_*v*_
	*β*_1_	*β*_2_	*β*_3_	*β*_4_	*β*_5_	
N(stems/ha)	Elev_CV_m	Elev_P75_m	Elev_P80_v	A_P30	Elev_P99_v	98.06
V(m^3^/ha)	Slope_s	Slope_m	A_P60			384.07
BA (m^2^/ha)	all_1st_co_2m_m	Elev_CV_v	Elev_max_v	Slope_m	Slope_s	257.74
QMD (cm)	Elev_P01_v	Slope_m	Slope_s	A_P10	A_P20	9.51
H_m_ (m)	all_1st_co_2m_m	Elev_CV_v	A_P10	A_P20	A_P30	4.66
H_lor_ (m)	Elev_ave_sq_m	Perc_W				7.03

The error variance of the MU volume was proportional to a power of the mean elevation of the point cloud, a model similar to that of [9,19–21]. Our results confirm that, for volume, residuals from LiDAR models show patterns of heteroscedasticity that can be successfully modeled assuming that the error variance is a relatively simple function of the mean elevation of a LiDAR or a photogrammetric point cloud. Expressions for the mean squared error of the EBLUP used in this study are valid when *η*, the parameter that defines the weight in the expression of the variance, is known. However, in practice, this parameter is unknown and treating it as fixed results in underestimation of the *mse*. Obtaining expressions for approximately unbiased *mse* estimators in a frequentist framework when *η* is unknown would require new developments in small area estimation theory, although parametric bootstrap or Bayesian methods could be used to overcome this problem.

For unit level models, the distribution of residuals and random effects showed departures from normality and tails heavier than expected for a normal distribution. Alternative estimators that are robust to outliers have been proposed in the literature [22]. While those types of estimators are out of the scope of this manuscript, it is important to note that the EBLUPs for unit level models are robust to outliers in the random effects [22]. Mixed models with uncertain random effects (i.e. the distribution of the random effects follows a mixture model, where the random effects are 0 with probability *π* and normally distribution with probability 1 − *π*) are an alternative solution that has received significant attention in the small area estimation literature recently [23,24], and can be a potential solution for modelling the distributions of random effects similar to those observed here.

### 3.2 Uncertainty of estimates

**Fig 2:**
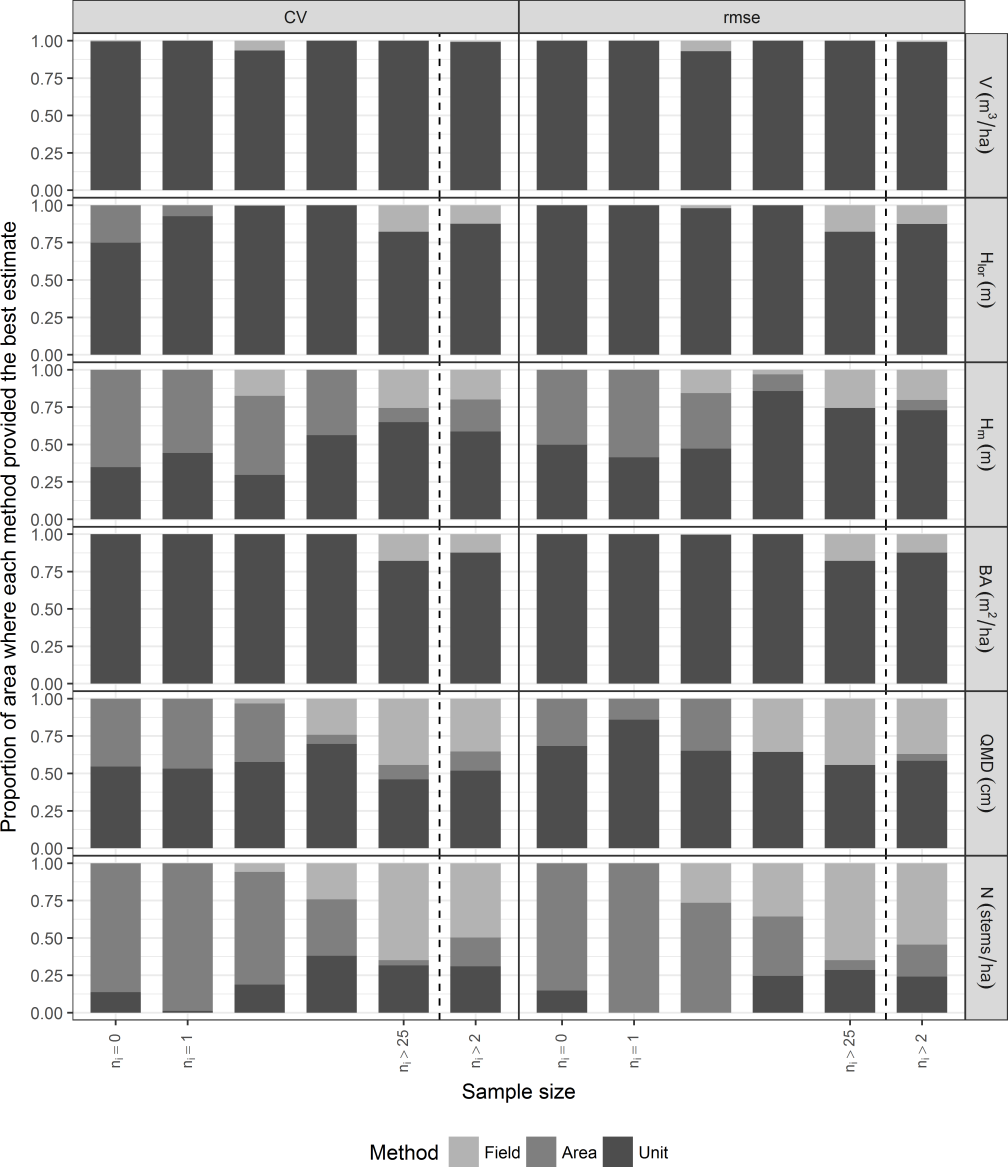
Proportion of area where each method was the best by sample size group. The group *n*_*i*_>2 includes all management units for which estimates and measures of uncertainty are available for the three methods.

For volume, basal area and Lorey’s height, EBLUPs based on unit level models had the lowest *rmse* and coefficient of variation for most or all MUs (Fig 2). For those variables, estimates based on either area level models or field data only resulted in the lowest *rmse* or coefficient of variation in a small fraction of the study area (less than 25% of the total area). For mean height, unit level models dominated the other methods, but the proportion of area where either area level models or field only estimates were best was clearly larger than for volume, Lorey’s height and mean height (Fig 2). A similar pattern was observed for QMD, although field based estimates were best for an area significantly larger than that where area level estimates were best. For stand density, area level models provided better estimates than any other method (Fig 2) and dominated in approximately 50% of the study area, but the difference in performance between area level and unit level models was small. This result is in accordance with previous studies where stand density has been predicted with poor accuracies when using LiDAR (e.g. [10,25]).

The ratios between the *rmse* estimated from the area- and unit level methods (Eq (5)), averaged across all MUs (weighted by MU area) (Table 3) showed that, for volume, predictions from the unit level model were much more precise than those from the area level model, and the latter were not particularly accurate for this variable. On average, the *rmse* of area level models was 2.83 times the *rmse* for unit level models. This ratio was always larger than 2 regardless of the number of plots in the MU. For N, BA, QMD, H_m_ and H_lor_, *rmse* for area level models was, on average, 1.43, 2.09, 1.40, 1.32 and 1.64 times greater than that for unit level models, respectively, and unit level models tended to dominate across all MU sizes. For all variables, unit level models performed better than area level models and direct estimators based on field plots alone, and area level models performed better than direct estimators. Thus, overall, incorporating auxiliary information, even when aggregated to the MU level, would improve estimation of forest variables for small areas (Table 3). The *rmse* for predictions of N,V, BA, QMD, H_m_ and H_lor_ from field measurements were, on average, 1.37, 1.45, 1.17, 1.26 and 1.3 times larger than those obtained using area level models (Table 3).

**Table 3:** Averages, weighted by MU area, for *rrmse*_ in Eqs (5), (6) and (7). The column method defines which method is in the numerator/denominator when computing the *rmse* ratios.

Variable	Methods	Averages (weighted by MU area) of *rmser*_					
		All MUs	MUs grouped by *n*_*i*_				
			0	1	2–5	6–25	>25
N(stems/ha)	Area/Unit	1.43	0.60	0.67	0.68	1.05	1.71
	Field/Area	1.37			4.70	1.84	0.61
	Field/Unit	1.29			2.71	1.45	0.97
V(m^3^/ha)	Area/Unit	2.83	2.21	2.17	2.00	2.20	3.18
	Field/Area	1.45			3.49	1.91	0.94
	Field/Unit	3.34			6.67	4.01	2.53
BA(m^2^/ha)	Area/Unit	2.09	2.30	2.45	1.96	2.01	2.13
	Field/Area	1.17			2.65	1.36	0.84
	Field/Unit	2.30			5.12	2.60	1.68
QMD(cm)	Area/Unit	1.40	1.12	1.13	1.06	1.23	1.52
	Field/Area	1.17			2.65	1.26	0.86
	Field/Unit	1.47			2.84	1.50	1.19
H_m_(m)	Area/Unit	1.32	1.05	1.09	1.00	1.17	1.44
	Field/Area	1.26			2.41	1.53	0.97
	Field/Unit	1.50			2.41	1.70	1.27
H_lor_ (m)	Area/Unit	1.64	1.18	1.19	1.24	1.37	1.81
	Field/Area	1.38			2.87	1.71	1.01
	Field/Unit	2.13			3.56	2.36	1.80

Considering the better performance of unit level models and the relatively low cost (around 1000 US$) of handheld GPS units capable of collecting phase observations and providing coordinates of similar accuracy to those obtained using geodetic devices when recording times are sufficiently large [26], unit level models may likely be the most sensible approach when other factors do not preclude using this methodology. However, the results of this study also suggest that area level EBLUPs improve forest inventories and can help in situations when unit level models cannot be applied. For example when using variable radius plots for fast field assessments, or when confidentiality restrictions, economic or operational constraints do not allow a precise plot positioning.

#### 3.2.1 Uncertainty of estimates by MU sample size

For the largest MUs the ratio of *rmse*_*Field i*_ to *rmse*_*Area i*_ in (6) is less than 1 for all variables except for H_lor_ where it takes the value 1.01 (Table 3). This indicates that, in the study area, for very large units with more than 25 plots, direct estimates outperform area level EBLUPs. When direct and unit level estimates are compared (7), the results show that differences become smaller as the sample size increase, but unit level models always remain superior to direct estimates (Table 3) except for N. For this variable, the ratio *rmse*_*Field i*_ to *rmse*_*Area i*_ becomes 0.97 which indicates that both field and unit level estimates are similarly accurate. In general, the inclusion of LiDAR auxiliary information through unit level models or through area level models allow for better estimates than when using only field data (Table 3); except for MUs with sample sizes larger than 25, where area level EBLUPs provide estimates with less accuracy than field estimates for all variables except Lorey’s Height. These results suggest that globally, area level models effectively improve results obtained from field only estimates.

The size of the MUs considered in this study was relatively large which might explain the relatively small gains obtained when using area level models in MUs containing more than 25 plots. Different results are possible in forest inventories at a smaller scale. In fact, in a study area with MUs of smaller size, the performance of these methods was better for several variables [6]. However the analysis of the accuracy of area level models for different inventory scales is a topic currently under research [27].

While prediction using LiDAR data in a unit level framework has been investigated during the last decades [10,28,29], very little is known about the performance of area level models. Results from this study provide a reference about the gain or loss of accuracy of using one type of EBLUP instead of another and, about how much each type of EBLUP reduces the uncertainty of estimates based only on field data. While, the comparisons made in this study are based on estimated root mean square errors, therefore subject themselves to some uncertainty, *rmse* estimators used in this analysis are unbiased, and values are reported by groups which favors the compensation of estimation errors. To the best of our knowledge the variance of the *rmse* estimators used in this analysis is not known and is a topic of clear interest for future research.

Another issue that may merit further examination is how the predictors for area level models are calculated when MUs are as large as here. In this study, we computed most area level predictors after aggregating the values of unit level predictors or DTM, DSM and DCHM rasters and computing their means and variances. Most of those predictors can be obtained using standard GIS arithmetic over raster layers, which is appealing for its simplicity. However, when proceeding in this way, predictors are calculated in a way that is not completely equal to the standard way of computing unit level predictors, in particular when computing quantities such as quantiles and variances. While computing predictors using the point clouds in the same way as it is done for unit level models is possible, the size of the MUs makes this an inoperative task. The size for 24 MUs was greater than 1000 ha (1090.54 ha to 12067.62 ha) and the MU shapes were fairly irregular. Calculating predictors in the same way as it was done for the unit level models would imply reading and dealing with 15 GB to 180 GB of point cloud information. The area level predictors could be directly computed from the LiDAR point clouds, which may result in better performance. Such analysis should be subject to future research.

## 4 Conclusions

Although differences for the *rmse* of estimates obtained by different methods depended greatly on the variable of interest, estimates with unit level models were, on average, much more accurate than those obtained using area level models. Area level estimates are consistently more precise than estimates obtained using only field information, and therefore, can enhance the results of traditional inventories when estimating for small areas. For areas with large sample sizes, direct design based estimators provide comparable accuracies without the need of making strong assumptions about the population of interest by assuming a model. Differences between methods tend to become smaller as the sample size increases, and for very large MUs area level models estimates can have larger *rmse* than traditional field only estimates.

From the results obtained in this analysis, we conclude that area level models improve forest inventories, however, their niche may be limited to situations where plot locations are not available or when using variable radius plots and MUs do not hold a large sample size that provides reliable estimates.

## Supporting information

S1 Appendix Computations of LIDAR predictors for unit level models and area level models. (DOCX)

S2 Appendix Unit level and area level models, EBLUPs and mean square error estimators. (DOCX)

S1 File Unit level dataset. (TXT)

S2 File Area level dataset. (TXT)
