# Supplementary material for: Analysis of area level and unit level models for small area estimation in forest inventories assisted with LiDAR auxiliary information
Source: PLoS One. 2017 Dec 7;12(12):e0189401. doi: 10.1371/journal.pone.0189401 (PMC5720784; doi:10.1371/journal.pone.0189401)
Supplement: S1 Appendix — (DOCX) [file pone.0189401.s001.docx]

# S1 Appendix. Computations of LIDAR predictors for unit level models and area level models.

1. LIDAR data

LIDAR data were collected during the spring and summer of 2008 and 2009 using a Leica LIDAR Phase II laser. The average return density was 8.1 returns/m^2^, the average flying altitude was 900m above sea level, and the field of view was 28° (±14° from nadir). Additional details about the LiDAR flight survey can be found in [1]. The LiDAR data was processed to obtain predictors for both unit and area level models.

1. Computation of unit level predictors

A grid with a cell size of 22.86 m (0.052ha) was overlaid on the study area and defined the population units. In total 44 predictors were obtained for each grid unit and field plot. These predictors were grouped depending on the data used for their computation.

1. Summaries of the LIDAR point clouds: These predictors were computed using FUSION [2] and are referred to as group 1 unit. In total, 28 predictors were derived directly from the LIDAR point clouds and included extreme values, percentiles, and fractions of pulses above different thresholds.
2. Predictors directly obtained from the Forest Operations Inventory (FOI) database. These predictors (group 2 unit) were the age and dominant species of the centroid of each field plot or grid unit according to the FOI database.
3. A third group of 14 predictors was obtained using a 1 m resolution, digital terrain model (DTM) and a 1 m resolution digital surface model (DSM). First, a digital canopy height model (DCHM) was obtained from the DTM and DSM. Then the DTM was used to derive 1 m resolution layers with slope and aspect. Finally, averages, standard deviations and ranges of the values of the DTM, DCHM, slope and aspect layers were calculated for each LiDAR grid unit and plot, which contained approximately 520 raster cells from the topographic layers. For the DTM, the min MU elevation was obtained. The aspect layer was also summarized by computing the percentage of the plot or LiDAR grid unit area facing North, East, South and West. This set of predictors is referred as group unit 3.

In Figure S 1, 1 a graphical summary of the computation of unit level predictors is presented. In Table S1 1, all predictors considered at the unit level are listed.


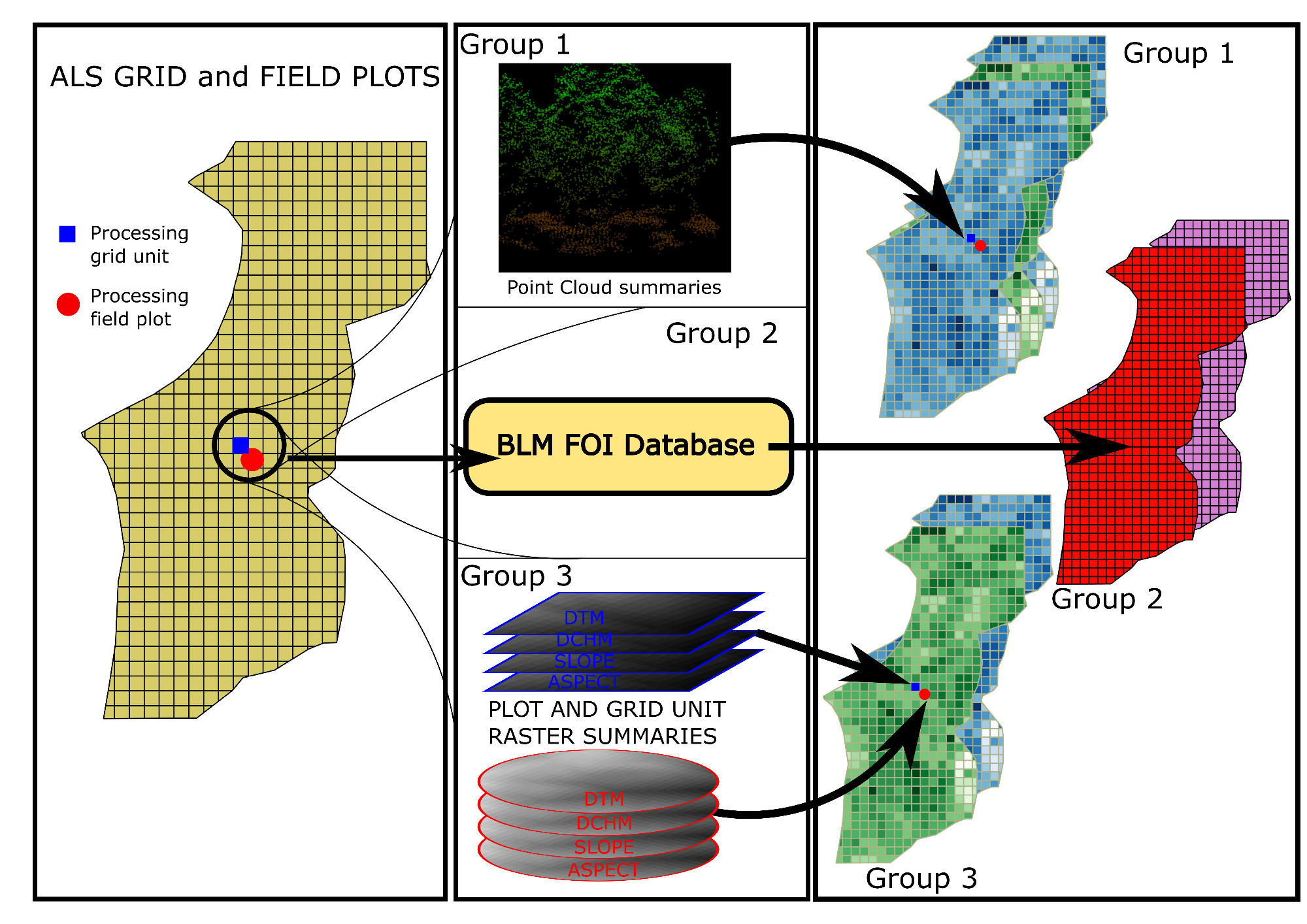


| Group | Source | | Variable | Abreviated name/s |
| --- | --- | --- | --- | --- |
| Group 1 unit | LIDAR point | | Percentage of returns above the mean return height | all_co_ab_mean |
|  |  |  | Percentage of returns above 2 m | all_co_ab_2m |
|  |  |  | Percentage of first returns above the mean return height | 1st_co_ab_mean |
|  |  |  | Percentage of first returns above 2 m | 1st_co_ab_2m |
|  |  |  | Number of first returns above mean/ Number of returns above the mean | all_1^st^_co_ab_mean |
|  |  |  | Number of first returns above 2m/ Number of returns above the 2m | all_1^st^_co_ab_2m |
|  |  |  | Return height percentiles (1th,5th, 10th, 20th,30th,40th,50th,60th,70th,75th,80th,90th,95th,99th) | Elev_P01, Elev_P05, Elev_P10, Elev_P20, Elev_P30, Elev_P40, Elev_P50, Elev_P60, Elev_P70, Elev_P75, Eleve_P80, Elev_P90, Elev_P95, Elev_P99 |
|  |  |  | Interquartile range of the return height distribution | Elev_IQ |
|  |  |  | Minimum return height | Elev_Min |
|  |  |  | Mean return height | Eev_Mean |
|  |  |  | Square of the mean return height | Elev_MeanSq |
|  |  |  | Max return height | Elev_Max |
|  |  |  | Standard deviation of the return heights | Elev_Stdv |
|  |  |  | Coefficient of variation of the return heights | Elev_CV |
|  |  |  | Average absolute deviation with respect to the mean return height | Elev_AAD |
| Group 2 unit | BLM forest inventory and operations database | | Age | Age |
|  |  |  | Dominant species | Spc (Categorical variable with three groups: Douglas-fir, mixed-conifer and hardwood) |
| Group 3 unit | 1 m celsize DTM & DSM (DTM and DSM were also obtained from the LIDAR point cloud) | DTM | Plot/grid unit DTM Min | DTM_Min |
|  |  |  | Plot/grid unit DTM Mean | DTM_Mean |
|  |  |  | Plot/grid unit DTM Range | DTM_Range |
|  |  |  | Plot/grid unit DTM Standard deviation | DTM_Stdv |
|  |  | DCHM (DSM-DTM) | Plot/grid unit DCHM Mean | DCHM_Mean |
|  |  |  | Plot/grid unit DCHM Range | DCHM_Range |
|  |  |  | Plot/grid unit DCHM Standard deviation | DCHM _Stdv |
|  |  | Slopes | Plot/grid unit Slope Mean | Slope_Mean |
|  |  |  | Plot/grid unit Slope Range | Slope_Range |
|  |  |  | Plot/grid unit Slope Standard deviation | Slope_Stdv |
|  |  | Aspects | Percentage of sample plot/grid unit area facing N | Perc_N |
|  |  |  | Percentage of sample plot/grid unit area facing S | Perc_S |
|  |  |  | Percentage of sample plot/grid unit area facing E | Perc_E |
|  |  |  | Percentage of sample plot/grid unit area facing W | Perc_W |

1. Area level predictors

For the area-level models, 87 predictors groups into four sets were calculated. The first two groups (group 1 area and group 2 area) were computed using the unit level predictors of group 1 unit, the last two groups (group 3 area and group 4 area) are equivalent to group 2 unit and group 3 unit.

- Group 1 area consisted on MU means and variances of all predictors in group 1 unit. This group contained 56 predictors.
- Group 2 area consisted of approximate percentiles of the LIDAR return height distribution within each MU. The return height distribution for the MU was approximated by first interpolating the return height distribution of each grid unit. Then distributions of all LiDAR grid units in a MU were aggregated to obtain the MU return height distribution. For this step the grid unit distributions obtained in the previous step were aggregated at the MU level weighting each grid unit distribution by its corresponding number or returns. Finally, the 1^st^, 5^th^, 10^th^, 20^th^, 25^th^, 30^th^, 40^th^, 50^th^, 60^th^, 70^th^, 75^th^, 80^th^ 90^th^, 95^th^ and 99^th^ percentile of the MU return height distribution were obtained for each MU. Figure S1 2 presents the logic for the computation of these approximated percentiles using as an example a hypothetical MU with only two grid units.
- Group 3 area consist of the age and dominant species of each MU according to the FOI database.
- Group 4 area is equivalent to group 1 unit, but in this case the 1 m resolution DTM, DCHM, slope and aspect layers were summarized by MU.


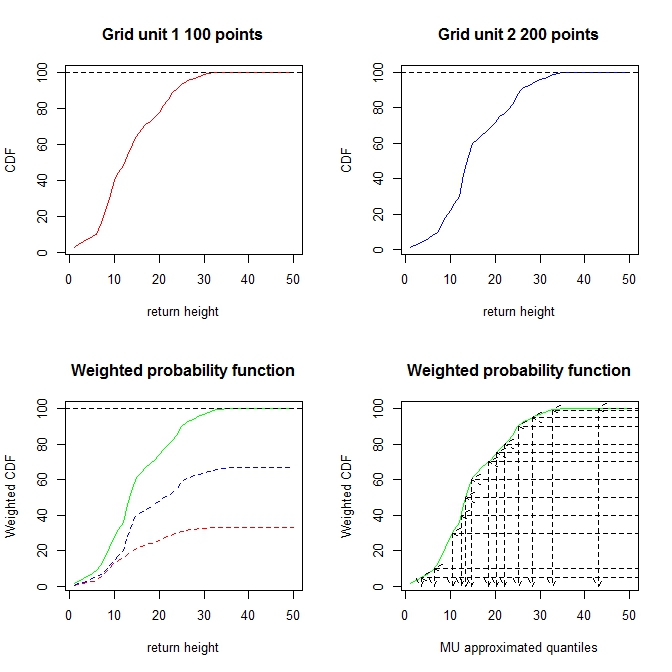


In Figure S 1, 3 a graphical summary of the computation of area level predictors is presented. In Table S1 2, lists all predictors at the area level.


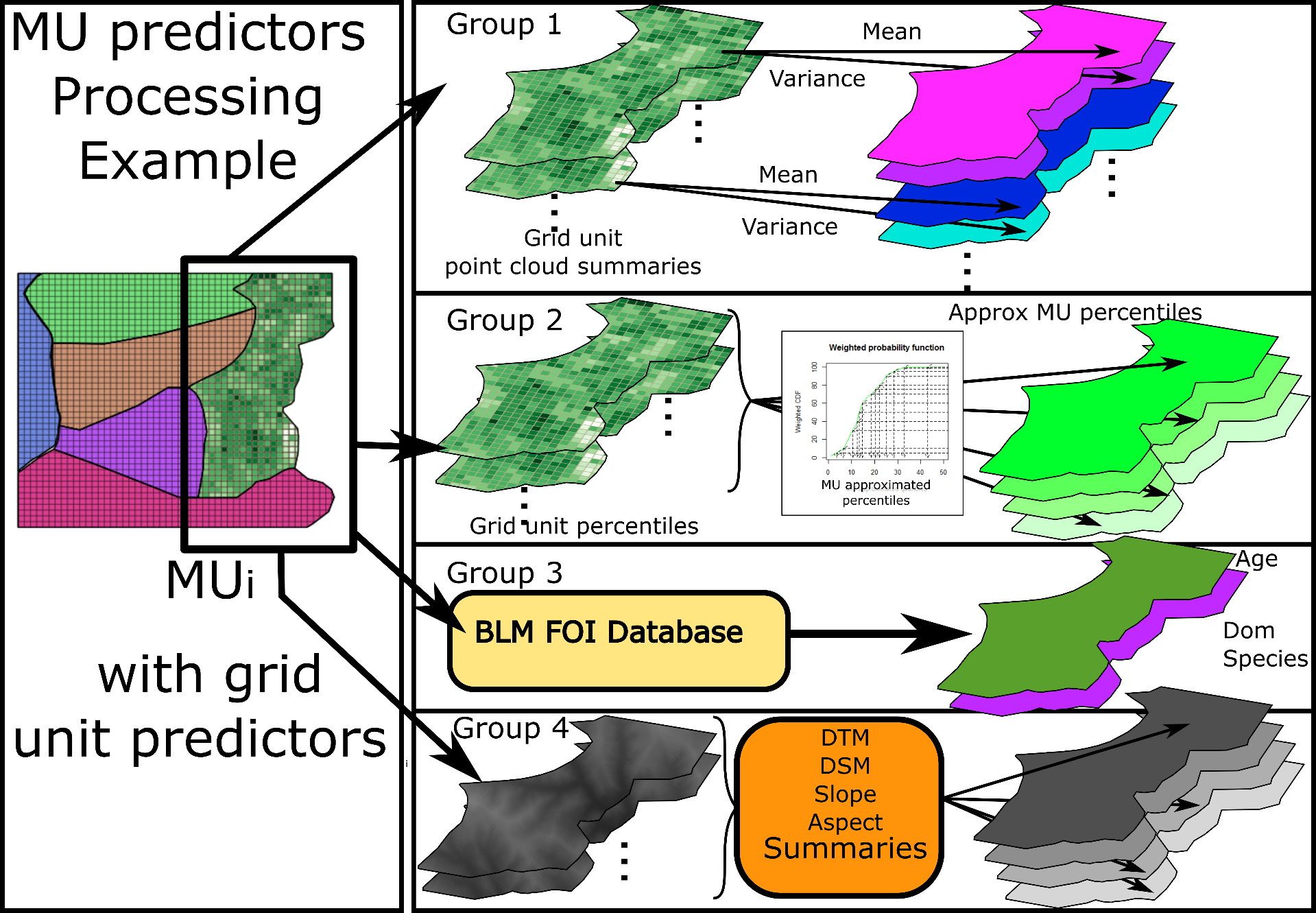


| Group | Source | | Variable | Abreviated name/s | |
| --- | --- | --- | --- | --- | --- |
| Group 1 area | LIDAR data | MU means and variances of unit level LIDAR point cloud predictors |  | Means (_m) | Variances (_v) |
|  |  |  | Mean and Variance of the grid unit values for Percentage of returns above the mean return height | all_co_ab_mean_m | all_co_ab_mean_v |
|  |  |  | Mean and Variance of the grid unit values for Percentage of returns above 2 m | all_co_ab_2m_m | all_co_ab_2m_v |
|  |  |  | Mean and Variance of the grid unit values for Percentage of first returns above the mean return height | 1st_co_ab_mean_m | 1st_co_ab_mean_v |
|  |  |  | Mean and Variance of the grid unit values for Percentage of first returns above 2 m | 1st_co_ab_2m_m | 1st_co_ab_2m_v |
|  |  |  | Mean and Variance of the grid unit values for Number of first returns above mean/ Number of returns above the mean | all_1st_co_ab_mean_m | all_1st_co_ab_mean_v |
|  |  |  | Mean and Variance of the grid unit values for Number of first returns above 2m/ Number of returns above the 2m | all_1st_co_ab_2m_m | all_1st_co_ab_2m_v |
|  |  |  | Mean and Variance of the grid unit values for Return height percentiles (1th,5th, 10th, 20th, 30th, 40th, 50th, 60th, 70th, 75th, 80th, 90th, 85th, 99th) | Elev_P01_m, Elev_P05_m, Elev_P10_m, Elev_P20_m, Elev_P30_m, Elev_P40_m, Elev_P50_m, Elev_P60_m, Elev_P70_m, Elev_P75_m, Eleve_P80_m, Elev_P90_m, Elev_P95_m, Elev_P99_m | Elev_P01_v, Elev_P05_v, Elev_P10_v, Elev_P20_v, Elev_P30_v, Elev_P40_v, Elev_P50_v, Elev_P60_v, Elev_P70_v, Elev_P75_v, Elev_P80_v, Elev_P90_v, Elev_P95_v, Elev_P99_v |
|  |  |  | Mean and Variance of the grid unit values for Interquartile range of the return height distribution | Elev_IQ_m | Elev_IQ_v |
|  |  |  | Mean and Variance of the grid unit values for Minimum return height | Elev_Min_m | Elev_Min_v |
|  |  |  | Mean and Variance of the grid unit values for Mean return height | Eev_Mean_m | Eev_Mean_v |
|  |  |  | Mean and Variance of the grid unit values for Square of the mean return height | Elev_MeanSq_m | Elev_MeanSq_v |
|  |  |  | Mean and Variance of the grid unit values for Max return height | Elev_Max_m | Elev_Max_v |
|  |  |  | Mean and Variance of the grid unit values for Standard deviation of the return heights | Elev_Stdv_m | Elev_Stdv_v |
|  |  |  | Mean and Variance of the grid unit values for Coefficient of variation of the return heights | Elev_CV_m | Elev_CV_v |
|  |  |  | Mean and Variance of the grid unit values for Average absolute deviation with respect to the mean return height | Elev_AAD_m | Elev_AAD_v |
| Group 2 area |  | Approximated percentiles | Approximated (1th,5th, 10th, 20th,30th,40th,50th,60th,70th,75th,80th,90th,85th,99th) percentiles | A_P01, A_P05, A_P10, A_P20, A_P25, A_P30, A_P40, A_P50, A_P60, A_P70, A_P75, A_P80, A_P90, A_P95, A_P99 | |
| Group 3 area | BLM forest inventory and operations database | | Age | Age | |
|  |  |  | Dominant species | Spc (Categorical variable with three groups: Douglas-fir, mixed-conifer and hardwood) | |
| Group 4 area | 1 m cell size DTM & DSM (DTM and DSM were also obtained from the LIDAR point cloud) | DTM | MU DTM Min | DTM_Min | |
|  |  |  | MU DTM Mean | DTM_Mean | |
|  |  |  | MU DTM Range | DTM_Range | |
|  |  |  | MU DTM Standard deviation | DTM_Stdv | |
|  |  | DCHM (DSM-DTM) | MU DCHM Mean | DCHM_Mean | |
|  |  |  | MU DCHM Range | DCHM_Range | |
|  |  |  | MU DCHM Standard deviation | DCHM _Stdv | |
|  |  | Slope | MU Slope Mean | Slope_Mean | |
|  |  |  | MU Slope Range | Slope_Range | |
|  |  |  | MU Slope Standard deviation | Slope_Stdv | |
|  |  | Aspect | Percentage of MU area facing N | Perc_N | |
|  |  |  | Percentage of MU area facing S | Perc_S | |
|  |  |  | Percentage of MU area facing E | Perc_E | |
|  |  |  | Percentage of MU area facing W | Perc_W | |
