# Supplementary material for: Analysis of area level and unit level models for small area estimation in forest inventories assisted with LiDAR auxiliary information
Source: PLoS One. 2017 Dec 7;12(12):e0189401. doi: 10.1371/journal.pone.0189401 (PMC5720784; doi:10.1371/journal.pone.0189401)
Supplement: S2 Appendix — (DOCX) [file pone.0189401.s002.docx]

# S2 Appendix. Unit level and area level models, EBLUPs and mean square error estimators.

Details about the EBLUPs for unit level models and area level models as well as their associated mean square error estimators are provided in the sections below. To differentiate predictors for unit level models and predictors for area level models, the former will be denoted with the letter $w$ instead of the usual $x$.

# S2.1. Unit level models

The general form of the unit level models was (equation (1)):

|  | $y_{ij}=\boldsymbol{x}_{ij}^{t}\boldsymbol{\beta}+v_{i}+e_{ij}$ | (1) |
| --- | --- | --- |

where the subscript $i=1,\ldots, m$($m$=84) indexes the MU and $j=1,\ldots,N_{i}$ indexes the population unit (grid cells or plots) within the MU. The terms $y_{ij}$, $\boldsymbol{x}_{ij}^{t}$ and $e_{ij}$ are the value of the response variable, the transpose of the vector of predictors, and the model errors for the $j^{th}$ population unit within the $i^{th}$ MU. The MU random effects are assumed to be independently distributed $v_{i}\sim N(0,\sigma_{v}^{2})$ and independent of the model errors $e_{ij}$. Heteroscedasticity of model errors was allowed, assuming that $e_{ij}={\sigma_{e}mcp}_{ij}^{\eta}\epsilon_{ij}$, where ${mcp}_{ij}$is the predictor most correlated to the variable of interest. Under this formulation, $\epsilon_{ij}$ is the standardized error, distributed as $\epsilon_{ij}\sim N\left( 0,1 \right)$, so that ${V\left( e_{ij} \right)}^{1/2}=\sigma_{e0}{mcp}_{ij}^{\eta}$, and $\eta$ is a model parameter with possible values equal to 0, 0.5 and 1. When heteroscedasticity was not present, the parameter $\eta$ was 0 and the term ${mcp}_{ij}^{\eta}$ was 1.

Model (1) applies to both the field sample plots and to the grid cells within the area of interest ($i$) for which predictions are sought. For an arbitrary subset of units, $\xi$, containing field plots and/or grid cells, model (1) can be expressed in matrix notation as

|  | $\boldsymbol{y}_{\xi}=\boldsymbol{X}_{\boldsymbol{\xi}}\boldsymbol{\beta}+\boldsymbol{Z}_{\xi}\boldsymbol{v}+\boldsymbol{\varepsilon}_{\boldsymbol{\xi}}$ | (2) |
| --- | --- | --- |

Where $\boldsymbol{y}_{\xi}$**,** $\boldsymbol{\varepsilon}_{\xi}$, and $\boldsymbol{X}_{\boldsymbol{\xi}}$ are obtained by respectively stacking the measurements of the variable of interest $y_{ij}$, the model errors $\varepsilon_{ij}$, and the transposed vector of predictors $\boldsymbol{x}_{\boldsymbol{i j}}^{\boldsymbol{t}}$ for all the $N_{\xi}$population units contained in the subset $\xi$. The term $\boldsymbol{v}=(v_{1},v_{2},\cdots v_{m})^{t}$ is the vector of MU random effects and $\boldsymbol{Z}_{\xi}$ is an incidence matrix with $N_{\xi}$ rows and $m$ columns, and where the element $z_{rc}=1$ if the $r^{th}$ unit belongs to the $c^{th}$ MU and 0 otherwise. The distribution of $\boldsymbol{v}$ is multivariate normal ${MVN(\boldsymbol{0}}_{m},\boldsymbol{G}\left( \sigma_{v}^{2} \right))$ with mean a vector of zeros of length $m$, and covariance matrix $\boldsymbol{G}\left( \sigma_{v}^{2} \right)=\sigma_{v}^{2}\boldsymbol{I}_{m}$, where $\boldsymbol{I}_{m}$ is the identity matrix of dimension $m$. The error term follows a multivariate normal distribution ${MVN(\boldsymbol{0}}_{N_{\xi}},\boldsymbol{R}_{\xi}\left( \sigma_{e}^{2} \right))$ where the covariance matrix, $\boldsymbol{R}_{\xi}\left( \sigma_{e}^{2} \right)$, is a $N_{\xi}$x$N_{\xi}$ block diagonal matrix with a block for each MU in $\xi$. The $r^{th}$ element of the diagonal of $\boldsymbol{R}_{\xi}\left( \sigma_{e}^{2} \right)$ is ${\sigma_{e}^{2}mcp}_{r}^{2\eta}$, being ${mcp}_{r}$ the value for the for the $r^{th}$ element of $\xi$ of the predictor most correlated to the variable of interest. Note that $\boldsymbol{R}_{\xi}$ is a function of both $\sigma_{e}^{2}$ and $\eta$. However, when estimating the $mse$, we will assume that $\eta$ is fixed and known and, therefore, will write $\boldsymbol{R}_{\xi}$ as a function of $\sigma_{e}^{2}$. Finally, the errors and random effects are assumed to be independent. Under these assumptions, $\boldsymbol{y}_{\xi}$ follows a multivariate normal distribution $MVN(\boldsymbol{X}_{\boldsymbol{\xi}}\boldsymbol{\beta},\boldsymbol{V}_{\xi}\left( \boldsymbol{\delta} \right)\boldsymbol{)}$ where $\boldsymbol{\delta=(}\sigma_{e}, \sigma_{v})$ is a vector of variance parameters and $\boldsymbol{V}_{\xi}\left( \boldsymbol{\delta} \right)=\boldsymbol{R}_{\xi}\left( \sigma_{e}^{2} \right)+\boldsymbol{Z}_{\xi}\boldsymbol{G}\left( \sigma_{v}^{2} \right)\boldsymbol{Z}_{\xi}^{t}$ is the covariance matrix. Note that $\boldsymbol{R}_{\xi}\left( \sigma_{e}^{2} \right)$ and $\boldsymbol{V}_{\xi}\left( \boldsymbol{\delta} \right)$ also depend on the values of $mcp$ and $\eta$, however $mcp$ is known and $\eta$ is assumed to be a fixed value. To simplify the notation, we only indicate the dependence of $\boldsymbol{R}_{\xi}\left( \sigma_{e}^{2} \right)$ and $\boldsymbol{V}_{\xi}\left( \boldsymbol{\delta} \right)$ on the unknown variance components.

Two different subsets of units and the accompanying sub-indexes will be systematically used throughout the manuscript. The subset $s$ will indicate the sample, the subset $i$, will refer to the $i^{th}$ management unit or area of interest.

## S2.1.1 EBLUP based on unit level mixed models and $\boldsymbol{mse}$ estimators.

In small area estimation problems, the parameter of interest is typically the mean value of the variable of interest in the considered MUs. Under model (1), this mean is the average of the values for all the grids cell in the area of interest [1]:

|  | $\mu_{i}=\frac{1}{N_{i}}\sum_{j=1}^{N_{i}} \left( \boldsymbol{x}_{ij}\boldsymbol{\beta}+v_{i}\mathbf{+}e_{ij} \right)={\bar{\boldsymbol{x}}}_{\boldsymbol{i}}\boldsymbol{\beta}\mathbf{+}\boldsymbol{m}_{i}^{t}\boldsymbol{v}+\frac{1}{N_{i}} \sum_{j=1}^{N_{i}} e_{ij}$ | (3) |
| --- | --- | --- |

where ${\bar{\boldsymbol{x}}}_{\boldsymbol{i}}$ is the MU average of the vectors $\boldsymbol{x}_{ij}$, $\boldsymbol{m}_{i}^{t}$ is a vector with the $i^{th}$ element equal to the number units that are within the MU ($N_{i}$) and, $\frac{1}{N_{i}}\sum_{j=1}^{N_{i}} e_{ij}$ is the average of the model errors within the MU. The MUs in this study area are large and contain a large number of population units. Then, $\frac{1}{N_{i}}\sum_{j=1}^{N_{i}} e_{ij}$ will be approximately 0 and we will not consider it in the remaining sections.

The EBLUP of $\mu_{i}$ under model (1) is a function of the sample data, because the sample is used to estimate $\hat{\boldsymbol{\beta}}$ and $\hat{\boldsymbol{\delta}}$, the vectors of the fixed effects and variance parameters. The vector of sample plot measurements (the sub index s indicates the sample) of the variable of interest is denoted as $\boldsymbol{y}_{\boldsymbol{s}}$, the matrix of predictors as $\boldsymbol{X}_{\boldsymbol{s}}$, and the covariance matrix of $\boldsymbol{y}_{\boldsymbol{s}}$ is denoted as $\boldsymbol{V}_{\boldsymbol{s}}\left( \boldsymbol{\delta} \right)$. Then, the predictor of $\mu_{i}$ is [2]:

|  | $\hat{\mu}_{Unit i}(\hat{\boldsymbol{\delta}})={\bar{\boldsymbol{x}}}_{i}\hat{\boldsymbol{\beta}}(\hat{\boldsymbol{\delta}})+\boldsymbol{m}_{i}^{t}\hat{\boldsymbol{v}}(\hat{\boldsymbol{\delta}})$ | (4) |
| --- | --- | --- |

where $\hat{\boldsymbol{\beta}}(\hat{\boldsymbol{\delta}})=\left\{ {\boldsymbol{X}_{\boldsymbol{s}}}^{\boldsymbol{t}}{\boldsymbol{V}_{\boldsymbol{s}}(\hat{\boldsymbol{\delta}})}^{-1}\boldsymbol{X}_{\boldsymbol{s}} \right\}^{-1}{\boldsymbol{X}_{\boldsymbol{s}}}^{\boldsymbol{t}}{\boldsymbol{V}_{\boldsymbol{s}}(\hat{\boldsymbol{\delta}})}^{-\mathbf{1}}\boldsymbol{y}_{\boldsymbol{s}}$ is the estimated vector of fixed parameters and $\hat{\boldsymbol{v}}(\hat{\boldsymbol{\delta}})= \boldsymbol{G}(\hat{\boldsymbol{\delta}}){\boldsymbol{Z}_{\boldsymbol{s}}}^{\boldsymbol{t}}{\boldsymbol{V}_{\boldsymbol{s}}(\hat{\boldsymbol{\delta}})}^{-\mathbf{1}}\{\boldsymbol{y}_{\boldsymbol{s}} - \boldsymbol{X}_{\boldsymbol{s}}\hat{\boldsymbol{\beta}}(\hat{\boldsymbol{\delta}})\}$ is the estimated vector of MU random effects. The sub index $Unit$ is used to differentiate the EBLUP for unit level models from the EBLUP for the area level models described in section 0 of the manuscript. For unsampled units the $\hat{\boldsymbol{v}}(\hat{\boldsymbol{\delta}})$ becomes 0 and $\hat{\mu}_{Unit i}(\hat{\boldsymbol{\delta}})$ is a synthetic predictor $\hat{\mu}_{Unit i}(\hat{\boldsymbol{\delta}})={\bar{\boldsymbol{x}}}_{i}\hat{\boldsymbol{\beta}}(\hat{\boldsymbol{\delta}})+\boldsymbol{m}_{i}^{t}$.

|  | $\hat{\mu}_{synthetic Unit i}(\hat{\boldsymbol{\delta}})={\bar{\boldsymbol{x}}}_{i}\hat{\boldsymbol{\beta}}(\hat{\boldsymbol{\delta}})$ | (5) |
| --- | --- | --- |

When $\hat{\boldsymbol{\delta}}$ is obtained using maximum likelihood (ML) or restricted maximum likelihood (REML), an approximately unbiased estimator of the mean squared error of the EBLUP, with an approximation of order$o\left( {m_{sampled}}^{-1} \right)$, where $m_{sampled}$ is the number of sampled MUs (64 total), is [2]:

|  | $mse\left( \hat{\mu}_{Unit i} \right)=g_{Unit i 1}\left( \hat{\boldsymbol{\delta}} \right)+ g_{Unit i 2}\left( \hat{\boldsymbol{\delta}} \right)+{2g}_{Unit i 3}\left( \hat{\boldsymbol{\delta}} \right)$ | (6) |
| --- | --- | --- |

Where $g_{Unit i 1}\left( \hat{\boldsymbol{\delta}} \right)$ represents the uncertainty of the estimation when both $\boldsymbol{\beta}$ and $\boldsymbol{\delta}$ are assumed to be known and its general expression is indicated in equation (7).

|  | $g_{Unit i 1}\left( \hat{\boldsymbol{\delta}} \right)=\boldsymbol{m}_{i}^{t}\boldsymbol{G}\left( \hat{\boldsymbol{\delta}} \right)\boldsymbol{m}_{i}\boldsymbol{-}\boldsymbol{m}_{i}^{t}\boldsymbol{G}\left( \hat{\boldsymbol{\delta}} \right){\boldsymbol{Z}_{\boldsymbol{s}}}^{t}{\boldsymbol{V}_{\boldsymbol{s}}}^{-1}\left( \hat{\boldsymbol{\delta}} \right)\boldsymbol{Z}_{\boldsymbol{s}}\boldsymbol{G}\left( \hat{\boldsymbol{\delta}} \right)\boldsymbol{m}_{i}$ | (7) |
| --- | --- | --- |

The term $g_{Unit AOI 2}\left( \hat{\boldsymbol{\delta}} \right)$, equation (8), represents the increase in the uncertainty due to the estimation of $\boldsymbol{\beta}$**.** In equation (8), $\boldsymbol{d}_{i}^{\boldsymbol{t}}\left( \hat{\boldsymbol{\delta}} \right)={\bar{\boldsymbol{x}}}_{i}^{t}\mathbf{-}\boldsymbol{b}_{i}^{\boldsymbol{t}}{\left( \hat{\boldsymbol{\delta}} \right)\boldsymbol{X}}_{\boldsymbol{s}}$ and $\boldsymbol{b}_{i}^{\boldsymbol{t}}\left( \hat{\boldsymbol{\delta}} \right)\boldsymbol{=}\boldsymbol{m}_{i}^{\boldsymbol{t}}\boldsymbol{G}{(\boldsymbol{\delta})\boldsymbol{Z}_{\boldsymbol{s}}}^{\boldsymbol{t}}{\boldsymbol{V}_{\boldsymbol{s}}(\boldsymbol{\delta})}^{\boldsymbol{-1}}$.

|  | $g_{Unit i 2}\left( \hat{\boldsymbol{\delta}} \right)\boldsymbol{=}\boldsymbol{d}_{i}^{\boldsymbol{t}}\left( \hat{\boldsymbol{\delta}} \right)\left\{ {\boldsymbol{X}_{\boldsymbol{s}}}^{\boldsymbol{t}}{\boldsymbol{V}_{\boldsymbol{s}}\left( \hat{\boldsymbol{\delta}} \right)}^{\boldsymbol{-1}}\boldsymbol{X}_{\boldsymbol{s}} \right\}^{\boldsymbol{-1}}\boldsymbol{d}_{i}\left( \hat{\boldsymbol{\delta}} \right)$ | (8) |
| --- | --- | --- |

Finally, the term $g_{Unit i 3}\left( \hat{\boldsymbol{\delta}} \right)$, is necessary to correct for bias that results when the variance parameters $\boldsymbol{\delta}$ are estimated. The term $g_{Unit i 3}\left( \hat{\boldsymbol{\delta}} \right)$ for REML [3,4] is (9):

|  | $g_{Unit i 3}\left( \hat{\boldsymbol{\delta}} \right)=tr\left\{ \frac{\partial\boldsymbol{b}_{i}^{\boldsymbol{t}}\left( \hat{\boldsymbol{\delta}} \right)}{\partial\boldsymbol{\delta}}\boldsymbol{V}_{\boldsymbol{s}}\left( \hat{\boldsymbol{\delta}} \right){\frac{\partial\boldsymbol{b}_{i}^{\boldsymbol{t}}\left( \hat{\boldsymbol{\delta}} \right)}{\partial\boldsymbol{\delta}}}^{t}\bar{V}(\hat{\boldsymbol{\delta}}) \right\}$ | (9) |
| --- | --- | --- |

Where $\bar{V}(\hat{\boldsymbol{\delta}})$, is the inverse of the Fisher information matrix for the unit level model (1) and $tr()$ is the operator that returns the trace of matrix. Details on $\bar{V}(\hat{\boldsymbol{\delta}})$ can be found in [2 pp 179-181]. The matrix $\boldsymbol{R}_{s}\left( \hat{\boldsymbol{\delta}} \right)$appears in equations (4), (7), (8) and (9) as it is part of $\boldsymbol{V}_{\boldsymbol{s}}\left( \hat{\boldsymbol{\delta}} \right)$. Previous studies [5–8]have shown that spatial correlation or model errors for variables like the ones we analyzed here disappears at distances in the range of 100 meters or less. Considering the spacing between plots, we will assume that errors of any pair of sample plots are uncorrelated so $\boldsymbol{R}_{s}\left( \hat{\boldsymbol{\delta}} \right)$ is a diagonal matrix.

For unsampled units, we estimate the parameter of interest and its mean square error using formulas (4),(6),(7),(8) and (9). We are implicitly assuming that model (1), holds for all MUs so $\boldsymbol{G}\left( \sigma_{v}^{2} \right)=\sigma_{v}^{2}\boldsymbol{I}_{m}$ and the incidence matrices $\boldsymbol{Z}_{\xi}$, that always have 84 columns, include all MUs, not only the 64 that were sampled. This is an assumption that we cannot test due to the lack of field information in these MUs, however, it seems a reasonable assumptions, provided that the unsampled MUs do not have ages or dominant species that would suggest that they were very different from other sampled MUs. Finally, using equation (4) for an unsampled unit is equivalent to using a synthetic estimator where $\hat{\mu}_{Unit i}(\hat{\boldsymbol{\delta}})={\bar{\boldsymbol{x}}}_{i}\hat{\boldsymbol{\beta}}(\hat{\boldsymbol{\delta}})$. For the mean square error the terms $g_{Unit i 2}\left( \hat{\boldsymbol{\delta}} \right)$ and $g_{Unit i 3}\left( \hat{\boldsymbol{\delta}} \right)$ remain the same as they relate to the uncertainty due to the estimation of $\boldsymbol{\beta}$ and $\boldsymbol{\delta}$, but (7) for unsampled areas becomes

|  | $g_{synthetic Unit i 1}\left( \hat{\boldsymbol{\delta}} \right)=\boldsymbol{m}_{i}^{t}\boldsymbol{G}\left( \hat{\boldsymbol{\delta}} \right)\boldsymbol{m}_{i}^{t}$ | (10) |
| --- | --- | --- |

as the term $\boldsymbol{-}\boldsymbol{m}_{i}^{t}\boldsymbol{G}\left( \hat{\boldsymbol{\delta}} \right){\boldsymbol{Z}_{\boldsymbol{s}}}^{t}{\boldsymbol{V}_{\boldsymbol{s}}}^{-1}\left( \hat{\boldsymbol{\delta}} \right)\boldsymbol{Z}_{\boldsymbol{s}}\boldsymbol{G}\left( \hat{\boldsymbol{\delta}} \right)\boldsymbol{m}_{i}^{t}$ will be always zero. If the vector $\boldsymbol{m}_{i}^{t}$ is kept constant, the effect of reducing the sample size is that $g_{Unit i 1}\left( \hat{\boldsymbol{\delta}} \right)$ becomes larger as the quantity that we substract to $\boldsymbol{m}_{i}^{t}\boldsymbol{G}\left( \hat{\boldsymbol{\delta}} \right)\boldsymbol{m}_{i}^{t}$, becomes smaller, becoming zero for unsampled MUs (10).

The root mean square error and coefficient of variation for the unit level models were computed as $rmse\left( \hat{\mu}_{Unit i} \right)=\sqrt{mse\left( \hat{\mu}_{Unit i} \right)}$ and $CV\left( \mu_{Unit i} \right)=\frac{rmse\left( \hat{\mu}_{Unit i} \right)}{\hat{\mu}_{Unit i}}$, respectively.

## S2.1.2. Model selection for unit level models

To select a parsimonious model for each variable of interest, we first selected the best linear fixed effect models including one to five predictors, without considering heteroscedasticity, for a total of 25 candidate models. We selected the models based on the adjusted R^2^, and plotted the adjusted R^2^ against the number of predictors. For all variables, the adjusted R^2^ rapidly increased when increasing the number of predictors and then reached a plateau where it only marginally improved when including additional predictors. A similar pattern of improvement was observed for other statistics such as BIC and model RMSE. For each candidate model, we added MU random effects and a weight with exponent $\eta$, which was allowed to take the values 0, 0.5 and 1, resulting in 3 models per candidate. For each triplet of models including the same fixed-effect predictors we visually inspected plots of Pearson’s standardized residuals vs predicted values, predicted vs observed values, quantile-quantile plots for the residuals and quantile-quantile plots for the estimated MU random effects. We selected the simplest model among those for which adding additional variables did not improve the adjusted R^2^, Pearson’s standardized residuals were uniformly spread, and residuals and the adjusted random effects did not show important departures from normality. All models were fitted using restricted maximum likelihood (REML). Once a model was selected for each variable of interest, the EBLUP and the $mse$ estimators were computed for all grid units and for all MUs in the study area.

# S2.2. Area level models

The basic Area level model, also known as Fay-Herriot model [9], arises from the combination of a sampling model and a regression model linking the parameter of interest for each MU to the predictors. The parameter of interest for the $i^{th}$ MU is the mean of the variable of interest, denoted as $\mu_{i}$. It is linked to the auxiliary information through the regression model

|  | $\mu_{i} =\boldsymbol{w}_{i}^{t}\boldsymbol{\beta}+v_{i}$ | (11) |
| --- | --- | --- |

where $v_{i}$ is assumed to be normally distributed with mean 0 and variance $\sigma_{v}^{2}$ and $\boldsymbol{w}_{i}$ is a vector of predictors computed at the MU level.

A direct estimator of $\mu_{i}$, based on the sample of plots within the MU, is available and it is denoted as $\hat{\mu}_{Field i}$. The subscript Field is used to stress the fact that the direct estimator is computed using only field information and no auxiliary information is considered for its computation. Both $\mu_{i}$ and $\hat{\mu}_{Field i}$ are related through the sampling model

|  | $\hat{\mu}_{Field i}=\mu_{i}+e_{i}$ | (12) |
| --- | --- | --- |

Where $e_{i}$ is the error of the direct estimator $\hat{\mu}_{Field i}$ for the $i^{th}$ MU, which is assumed to be normally distributed with mean 0 and variance $\sigma_{e i}^{2}$. The direct estimator $\hat{\mu}_{Field i}$ in this case is the sample mean of the variable of interest for the field plots within the $i^{th}$ MU. When the sampling model and the regression model are combined, we obtain model (13).

|  | $\hat{\mu}_{Field i} =\boldsymbol{w}_{i}^{t}\boldsymbol{\beta}+v_{i}+e_{i}$ | (13) |
| --- | --- | --- |

## S2.2.1 EBLUP based on area level models and $\boldsymbol{mse}$ estimators.

The R package sae [10] and their functions eblupFH and mseFH were used to estimate $\boldsymbol{\beta}$ and $\sigma_{v}^{2}$ using REML, and to compute the EBLUP (14) and its estimated $mse$ for each MU:

|  | $\hat{\mu}_{Area i}=\boldsymbol{w}_{i}^{t}\hat{\boldsymbol{\beta}}+\gamma_{i}(\hat{\mu}_{Field i}-\boldsymbol{w}_{i}^{t}\hat{\boldsymbol{\beta}})$ | (14) |
| --- | --- | --- |

Where $\gamma_{i}=\frac{\hat{\sigma}_{v}^{2}}{\hat{\sigma}_{v}^{2}+\sigma_{e i}^{2}}$ determines the degree of shrinking towards the synthetic estimator$\boldsymbol{w}_{i}^{t}\hat{\boldsymbol{\beta}}$ and the variance estimates $\sigma_{e i}^{2}$ were input parameters that were treated as known. For MUs without any field plots, a direct estimator cannot be calculated, and when the MU sample size is smaller than two plots $\sigma_{e i}^{2}$ cannot be estimated. Thus, for MUs with less than two field plots (MUs with only one plot and unsampled MUs) it is necessary to rely on the synthetic estimator (15).

|  | $\hat{\mu}_{synthetic Area i}=\boldsymbol{w}_{i}^{t}\hat{\boldsymbol{\beta}}$ | (15) |
| --- | --- | --- |

A mean squared error estimator with order of approximation $o\left( {m_{s}}^{-1} \right)$ for $\hat{\mu}_{Area i}$ when $\hat{\sigma}_{v}^{2}$ is obtained using REML can also be expressed as the sum of three terms with similar interpretation as for the unit level models (16).

|  | $mse\left( \hat{\mu}_{Area i} \right)=g_{Area i 1}\left( \hat{\sigma}_{v}^{2} \right)+ g_{Area i 2}\left( \hat{\sigma}_{v}^{2} \right)+{2g}_{Area i 3}\left( \hat{\sigma}_{v}^{2} \right)$ | (16) |
| --- | --- | --- |

For the area level model (13, the expressions of $g_{Area 1}\left( \hat{\sigma}_{v}^{2} \right)$, $g_{Area 2}\left( \hat{\sigma}_{v}^{2} \right)$ and ${2g}_{Area 3}\left( \hat{\sigma}_{v}^{2} \right)$ are (17, (18 and (19):

|  | $g_{Area i 1}\left( \hat{\sigma}_{v}^{2} \right)=\gamma_{i}\sigma_{e i}^{2}$ | (17) |
| --- | --- | --- |
|  | $g_{Area i 2}\left( \hat{\sigma}_{v}^{2} \right)\boldsymbol{=}{\boldsymbol{(}1\boldsymbol{-}\gamma_{i}\boldsymbol{)}}^{\boldsymbol{2}}\boldsymbol{w}_{i}^{t}\left\{ \sum_{i:n_{i}>2} \frac{\boldsymbol{w}_{i}^{t}\boldsymbol{w}_{i}}{\hat{\sigma}_{v}^{2}+\sigma_{e i}^{2}} \right\}^{-1}\boldsymbol{w}_{i}$ | (18) |
|  | $g_{Area i 3}\left( \hat{\sigma}_{v}^{2} \right)=\sigma_{e i}^{4}{(\hat{\sigma}_{v}^{2}+\sigma_{e i}^{2})}^{-3}\bar{V}(\hat{\sigma}_{v}^{2})$ | (19) |

The summation on (18) only includes the MUs with at least two plots, and $\bar{V}(\hat{\sigma}_{v}^{2})$ is the inverse of the Fisher information matrix for model (13). Details on $\bar{V}(\hat{\sigma}_{v}^{2})$ can be found in [11]. As indicated in [5 p. 136] the $mse$ estimator of $\hat{\mu}_{synthetic i}$ used for MUs with less than two field plots is (20)

|  | $mse\left( \hat{\mu}_{synthetic Area i} \right)\boldsymbol{=}\boldsymbol{w}_{i}^{t}\left\{ \sum_{i:n_{i}>2} \frac{\boldsymbol{w}_{i}^{t}\boldsymbol{w}_{i}}{\hat{\sigma}_{v}^{2}+\sigma_{e i}^{2}} \right\}^{-1}\boldsymbol{w}_{i}+\hat{\sigma}_{v}^{2}$ | (20) |
| --- | --- | --- |

For the area level approach, the variance of the sampling error, $\sigma_{e i}^{2}$, is assumed to be known and $\sigma_{v}^{2}$ is estimated. Because in practical applications this is not the case, an estimator $\hat{\sigma}_{e i}^{2}= \frac{1}{n_{i}}\sum_{j=1}^{n_{i}} \frac{{(y_{i}-\hat{\mu}_{Field i})}^{2}}{n_{i}-1}$ of the variance $\sigma_{e,i}^{2}$ of the sample mean for each MU is computed. Then, the estimates of $\hat{\sigma}_{e i}^{2}$were smoothed using a generalized variance function (GVF) [12]. The GVF used in this study is indicated in equation (21)

|  | $\tilde{\sigma}_{e i}^{2} =\frac{\sigma_{e,0}^{2}}{n_{i}}$ | (21) |
| --- | --- | --- |

The parameter $\sigma_{e 0}^{2}$ was obtained regressing $\hat{\sigma}_{e i}^{2}$ against $\frac{1}{n_{i}}$ and the smoothed variances $\tilde{\sigma}_{e i}^{2}$ were plugged-in in estimators (14) and (16) replacing $\sigma_{e i}^{2}$.

The root mean square error and coefficient of variation for the unit level models were computed as $rmse\left( \hat{\mu}_{Area i} \right)=\sqrt{mse\left( \hat{\mu}_{Area i} \right)}$ and $CV\left( \mu_{Area i} \right)=\frac{rmse\left( \hat{\mu}_{Area i} \right)}{\hat{\mu}_{Area i}}$, respectively.

## S2.2.2. Model selection for area level models

The model selection procedure consisted of an exhaustive search where the best models with one through six predictors were obtained for each variable of interest. Considering model (11), only fixed effects models with constant variance were considered. The thirty candidates were sorted based on their adjusted R^2^. Quantile-quantile plots and residuals against fitted values plots were inspected to ensure normality and constant variance. The simplest model in the region where an increase in the number of predictors did not improved the adjusted R^2^, showing an approximately constant and normal residual variance was selected.

References

1. Mauro F, Molina I, García-Abril A, Valbuena R, Ayuga-Téllez E. Remote sensing estimates and measures of uncertainty for forest variables at different aggregation levels. Environmetrics. 2016;27: 225–238. doi:10.1002/env.2387

2. Rao JNK, Molina I. Empirical Best Linear Unbiased Prediction (EBLUP): Theory. Small Area Estimation. John Wiley & Sons, Inc; 2015. pp. 97–122. Available: http://dx.doi.org/10.1002/9781118735855.ch5

3. Datta GS, Lahiri P. A unified measure of uncertainty of estimated best linear unbiased predictors in small area estimation problems. Stat Sin. 2000;10: 613–628.

4. Das K, Jiang J, Rao JNK. Mean squared error of empirical predictor. Ann Stat. 2004;32: 818–840. doi:10.1214/009053604000000201

5. Breidenbach. J, Kublin E, McGaughey R, Andersen HE, Reutebuch S. Mixed-effects models for estimating stand volume by means of small footprint airborne laser scanner data. Photogramm J Finl. 2008;21: 4–15.

6. Rahlf J, Breidenbach J, Solberg S, Næsset E, Astrup R. Comparison of four types of 3D data for timber volume estimation. Remote Sens Environ. 2014;155: 325–333. doi:10.1016/j.rse.2014.08.036

7. Breidenbach J, McRoberts RE, Astrup R. Empirical coverage of model-based variance estimators for remote sensing assisted estimation of stand-level timber volume. Remote Sens Environ. 2016;173: 274–281. doi:10.1016/j.rse.2015.07.026

8. Mauro F, Monleon VJ, Temesgen H, Ruíz Fernández LÁ. Analysis of spatial correlation in predictive models of forest variables that use LiDAR auxiliary information. Can J For Res. 2017; doi:10.1139/cjfr-2016-0296

9. Fay RE, Herriot RA. Estimates of Income for Small Places: An Application of James-Stein Procedures to Census Data. J Am Stat Assoc. 1979;74: 269–277. doi:10.2307/2286322

10. Molina I, Marhuenda Y. sae: An R Package for Small Area Estimation. R J. 2015;7: 81–98.

11. Rao JNK, Molina I. Empirical Best Linear Unbiased Prediction (EBLUP): Basic Area Level Model. Small Area Estimation. John Wiley & Sons, Inc; 2015. pp. 123–172. Available: http://dx.doi.org/10.1002/9781118735855.ch6

12. Goerndt ME, Monleon VJ, Temesgen H. A comparison of small-area estimation techniques to estimate selected stand attributes using LiDAR-derived auxiliary variables. Can J For Res. 2011;41: 1189–1201.
